# Gaping gaps in rural mental health care: understanding causes and prioritizing solutions

**DOI:** 10.1192/j.eurpsy.2024.1232

**Published:** 2024-08-27

**Authors:** R. Gupta

**Affiliations:** Institute of Mental Health, Pt.BD Sharma University of Health Sciences, Rohtak, India

## Abstract

**Introduction:**

Mental health is crucial and is the backbone of all dimensions of health; physical, social and spiritual. Mental health has multiple interfaces and it is important to bring mental health to the center stage as it is the key regulator of all human activities. Unfortunately, there are alarming gaps in mental health care especially in rural areas which require attention of mental health professionals and policy makers.The study aims to understand the causes of these gaps and suggest possible and practical solutions to bridge them.

**Objectives:**

To study the spectrum of mental health gaps present in rural areas of Haryana, a state in the northern part of India and find culturally sensitive and relevant solutions keeping in view the socio economic realities and prevalent legal framework.

**Methods:**

Any factor having bearing on mental health but is operative sub-optimally would be considered as mental health gap for the current investigation. Rural camps were organized in 10 villages to assess the service gap at three different levels: overt (measurable), covert (including attitudinal) and ancillary (including those embedded in the psychiatry evaluation and treatment). The camps were organized by following these three basic steps: 1) Evaluating the geographic and demographic details of the villages selected. This was done by meeting the key stakeholders of the villages and the official health and service statistics available on the government website 2) Camp by multidisciplinary team in the villages with an advance intimation. The team members evaluated the mental health care awareness and the felt needs by interviewing all the villagers attending the camp on that particular day. 3) Post camp review by the team to analyze the service gaps and steps to address and narrow the gaps.

**Results:**

Apart from inadequate availability of professional and infrastructural resources, there were many  attitudinal and ancillary gaps serving as obstacles to treatment seeking. Trust gaps leading to poor acceptance and legislation not congruent with the socio cultural needs were key impediments. Rural people had more faith in Spiritual leaders and faith healers for their mental health issues and medical help was sought only when they have signs of physical illness. Mental health and illnesses were not on priority. Availability, accessibility and affordability of health services were important factors needing immediate attention.

**Conclusions:**

Rural services need to be augmented by de professionalization and task shifting is the key to address and cover the yawning gaps in the services. Massive, coordinated, multidisciplinary and sustainable efforts are needed to bridge the multitude of gaps keeping in view poverty and illiteracy as compounding factors.

**Disclosure of Interest:**

None Declared

